# Kidney cadmium levels and associations with urinary calcium and bone mineral density: a cross-sectional study in Sweden

**DOI:** 10.1186/1476-069X-12-22

**Published:** 2013-03-07

**Authors:** Maria Wallin, Gerd Sallsten, Elisabeth Fabricius-Lagging, Christian Öhrn, Thomas Lundh, Lars Barregard

**Affiliations:** 1 Department of Occupational and Environmental Medicine, Sahlgrenska University Hospital and Academy, University of Gothenburg, PO Box 414, SE-405 30, Gothenburg, Sweden; 2 Department of Nephrology, Sahlgrenska University Hospital, Gothenburg, Sweden; 3 Department of Nephrology, Boras Hospital, Boras, Sweden; 4 Department of Occupational and Environmental Medicine, Lund University Hospital, Lund, Sweden

**Keywords:** Bone, Bone mineral density, Cadmium, Kidney, Urinary calcium, Kidney donor

## Abstract

**Background:**

Cadmium (Cd) can cause renal damage and osteoporosis after high-level exposure. Recently such effects, including increased urinary excretion of calcium, have been shown also at low-level exposure, as measured by Cd in blood or urine. However, associations with kidney Cd have not been examined. The aim of this study was to explore the relation between kidney Cd and urinary calcium excretion, or bone mineral density.

**Methods:**

Cd was determined in kidney cortex biopsies from 109 living kidney donors. Serum was analyzed for ionized calcium, parathyroid hormone and vitamin D. Calcium was analyzed in overnight and 24-hour urine samples. Bone mineral density was measured in a subgroup of 67 donors. Associations between single variables were assessed by Spearman and Pearson correlation coefficients. Differences between independent groups were compared using Student’s t-test. For related samples, paired t-test was applied. Associations between urinary calcium and kidney Cd, ionized serum calcium, serum parathyroid hormone, inactive and active vitamin D and background variables were assessed using multiple linear regression and logistic regression.

**Results:**

In spite of relatively low kidney Cd levels (median 13 μg/g, range 1.5-55 μg/g) kidney Cd and urinary calcium were positively associated, mainly caused by an association in women. Donors with kidney Cd above the median (subgroup mean 23 μg/g) had significantly higher excretion of urinary calcium normalized for creatinine than those below the median (subgroup mean 7.3 μg/g). In women, also the excretion of Ca per hour was higher in those with high kidney Cd (24 hour sample mean 0.21 vs. 0.15 mmol/h; overnight sample 0.16 vs. 0.11 mmol/h). There were negative associations between kidney Cd and bone mineral density, most of which, however, disappeared in multivariate analyses.

**Conclusions:**

This study provides support for an association between kidney Cd levels and urinary calcium excretion in women, but not in men. The results strengthen the case for preventive measures against Cd pollution.

## Background

Cadmium (Cd) is a heavy metal, which occurs in the environment both naturally and as contamination from industries and agricultural fertilizers [1]. Cd is known to cause renal damage and bone demineralization and has also been classified as carcinogenic to humans [1]. The two major sources of Cd exposure in the general population are diet and tobacco smoking. Although women have lower energy intake and Cd intake than men, they usually have higher Cd concentrations in blood, urine and kidney, since iron deficiency, which is more common among women, is known to increase gastrointestinal absorption [2-5]. Cd is thought to use the same transport pathways as essential metals such as zinc, calcium and iron in the intestine, and also calcium deficiency might increase Cd uptake [6,7]. After absorption, Cd is transported in the blood to the liver where it forms a complex with metallothionein (MT). In the kidneys, this Cd-MT complex is filtered in the glomeruli and thereafter reabsorbed in the renal tubules [2]. Cadmium is then accumulated in the kidney cortex, where it has a biological half-life of 10-30 years [1,2,5]. Cadmium is toxic especially to the proximal renal tubules, but may also cause impaired glomerular filtration and even renal failure [1]. An early effect of tubular damage is increased excretion of low molecular weight proteins [2], resulting from compromised tubular reabsorption. Urinary cadmium (U-Cd) is often used as a measure of long-term exposure to Cd, as it is assumed to be proportional to kidney cadmium (K-Cd) and reflects the body burden, as long as the kidney function is intact [1,2,5].

The effects of Cd on bone were first reported from Japan, where Cd-polluted water was used on the rice fields and caused the Itai-Itai disease, a combination of osteomalacia, osteoporosis and kidney damage [2]. Recently, several studies have shown increased risk of osteoporosis and/or fractures even at low-level Cd exposure [8-13]. However, the mechanism for this effect of Cd on bone is not clear, despite several proposed theories, including interference with parathyroid hormone (PTH) or kidney enzymes involved in the activation of vitamin D [14,15]. Inactive vitamin D, 25(OH)D3, is hydroxylated in the kidney to its active form 1,25(OH)_2_D3, which increases the intestinal calcium absorption as well as the reabsorption of bone mineral matrix [14]. PTH has an important role in calcium homeostasis, as it stimulates vitamin D activation in the kidney, increases renal calcium reabsorption in the distal tubules and affects bone cells such as osteoblasts, osteoclasts and stromal cells [16].

Several studies have reported that occupational exposure to Cd increases the risk of kidney stones, which might be related to increased urinary calcium excretion [2]. Wu et al. reported a significant dose-response relationship between the prevalence of hypercalciuria and urinary Cd excretion in China [17]. Thus, one possible mechanism for the effects of Cd on bone is increased urinary calcium (U-Ca) excretion, caused by Cd-induced impairment of tubular reabsorption [2,18]. A study by Buchet et al. [19] showed that urinary calcium excretion, as well as other markers of renal tubular dysfunction, was significantly associated with Cd excretion in urine. However, recent studies indicate that increased calciuria might be caused by a direct effect of Cd on bone [5,12,20].

The main purpose of this study was to explore the relation between kidney Cd and urinary calcium excretion in the general population, testing the hypothesis that long-term low-level exposure to Cd can increase calcium excretion in urine. In order to avoid possible artifacts by comparing two urinary biomarkers in the same sample [21], we studied the association between kidney Cd and urinary calcium. In addition, we investigated the relation between kidney Cd and bone mineral density. This is the first study to use kidney biopsy data for cadmium analyses from living donors, who we assume represent the healthy part of the general population.

## Methods

### Kidney donors

At the Department of Transplantation and Liver Surgery at Sahlgrenska University Hospital (Gothenburg, Sweden), 167 living kidney donors were invited to the present study, performed between January 1999 and June 2002 and between April 2004 and February 2005. Of these 167 kidney donors, 152 accepted to participate in the study (81%). The median age of the 152 donors included in the study was 50 years (range 24-70). 87 of the included donors were women and 65 were men [22].

Before being accepted as kidney donors, the subjects had been examined with routine blood and urine tests, radiology and kidney function tests. These tests and examinations were made less than one year before the transplantation.

One or two days prior to transplantation, the donors were admitted to the hospital and underwent additional routine examinations as well as tests according to a study protocol. A physical examination was made and the donors were interviewed about their medical history. They also answered a questionnaire concerning occupation and smoking habits [22]. Blood tests were taken on the day of admission. A timed overnight urine sample was taken in the morning after admission and in most cases a separate timed 24-hour urine sample was also collected. Urine volume was measured in both samples. From April 2000 to December 2004, bone mineral density was measured in 95 of the donors.

A wedge biopsy was obtained from the kidney cortex during transplantation in 126/152 donors (83%) as part of the routine procedure at the Sahlgrenska University Hospital. The biopsy, which was taken from the lower pole of the kidney after revascularization, is normally used to determine the condition of the donor kidney and as a reference in case of later biopsies. Histopathological examination confirmed that the biopsy was taken from the kidney cortex. A part of the wedge biopsy was taken for analysis of heavy metal content. In 109 of the 126 donors (87%, 60/87 women and 49/65 men), this part of the biopsy was transferred into pre-weighed acid-washed glass tubes and then frozen. The remaining 17 parts were too small or of inadequate quality to be used for chemical analysis.

All subjects gave informed consent to their participation in the study, which was approved by the Ethics Committee at the University of Gothenburg.

### Urine, blood and kidney biopsy analyses

In the 109 donors with available kidney metal concentrations, 106 timed morning urine samples and 95 24-hour urine samples could be collected. The urine was analyzed for creatinine, using the Jaffé method (Roche Diagnostics) before June 2004 and thereafter with an enzymatic method (photometry, Modular P, Roche and CREAplus R1, R2, Roche Diagnostics, Mannheim, Germany), and calcium, using photometry (Modular, Roche and Ca R1, R2, Roche Diagnostics, Mannheim, Germany). Imprecision was 3-5% (CV, coefficient of variation). In the timed overnight sample, urinary calcium was missing in one sample. In 24 hour urine, urinary calcium was missing for two samples, and results were excluded for three samples as the sample time was too short (≤15 hours). The results for 24-hour urine samples with volumes <700 ml (n = 3) and >5000 ml (n = 1) were considered false or not representative and were excluded from the statistical analyses. Statistical analyses were made on the remaining 86 24-hour urine samples. Serum samples were analyzed for ionized Ca, parathyroid hormone (PTH) and inactive and active vitamin D3 (calcidiol or 25(OH)D3 and calcitriol or 1,25(OH)_2_D3). Ionized S-Ca (pH adjusted) was measured by ion-selective electrometry (ABL 805/825, Radiometer Copenhagen). PTH was measured by an immunoradiometric assay (IRMA, manual method) before November 2001 and thereafter by an immunochemical luminometric assay (Nichols Advantage Specialty System). Serum levels of 25(OH)D3 and 1,25(OH)_2_D3 were examined using a radioimmunochemical method (RIA, manual method, Perking Elmer Wizard 1470 gamma counter). All standard serum and urine analyses were performed by an ISO accredited laboratory (Department of Clinical Chemistry, Sahlgrenska University Hospital, Gothenburg). Cd concentrations in the kidney biopsies were determined by inductively coupled plasma-mass spectrometry (ICP-MS; Thermo X7, Thermo Elemental, Winsford, UK) [23], for details, see Barregard et al. [22].

### Assessment of BMD

BMD (bone mineral density; g/cm^2^) of the total body, lumbar spine, femoral neck, trochanter, ward and forearm was measured in a subgroup (67 of the 109 donors with data available for kidney Cd) using dual-energy X-ray absorptiometry (Lunar DPX-L, GE Lunar Corp.).

### Statistical analyses

Associations between single variables were assessed by Spearman and Pearson correlation coefficients (r_s_ and r_p_). Differences between groups were compared using Student’s t-test for independent groups and paired t-test for related samples. Associations between urinary calcium and kidney Cd, ionized serum calcium, serum PTH, inactive and active vitamin D and background variables were assessed using multiple linear regression and logistic regression. We chose to calculate urinary calcium excretion both per hour and normalized for creatinine. The reason for this is that we would like to compare urinary Ca levels in our study with other published studies which have used U-Ca normalized for creatinine (as samples were not timed). We also wanted to correct for the fact that males have higher lean body mass than females, and therefore excrete more Ca per hour. In order to detect a possible threshold effect of kidney Cd, we also calculated associations with urinary calcium after dividing the donors in two groups, with “high” or “low” kidney cadmium (K-Cd > or ≤ the median).

Since bone resorption increases after menopause in women, a menopause variable was included in the regression analyses, assuming menopause for women aged >51 years, which is the median age of natural menopause [24]. Statistical evaluations were performed using SAS (version 9.2).

## Results

A summary of Cd concentrations in the kidney cortex biopsies from the 109 donors and some biomarkers related to calcium metabolism are shown in Table 1. The Cd concentrations were generally relatively low (mean 15.0, median 12.9 μg/g wet weight). Women had significantly higher kidney Cd than men (mean 17.1, median 14.7 μg/g wet weight for women and mean 12.5, median 10.9 μg/g wet weight for men, p = 0.01).

**Table 1:** Kidney cadmium concentrations (K-Cd), calcium excretion (U-Ca) and levels of biomarkers related to calcium metabolism in healthy kidney donors

	**All**			**Men**			**Women**		
	**N**	**Median**	**Range**	**N**	**Median**	**Range**	**N**	**Median**	**Range**
K-Cd (μg/g wet weight)	109	12.9	1.5-55.4	49	10.9	1.6-31.7	60	14.7	1.5-55.4
Age (years)	109	51	24-70	49	52	32-70	60	50	24-64
Smokers: never/ever	41/68			19/30			22/38		
**24-hour sample**									
U-Ca (mmol/l)	86	3.0	0.74-7.1	39	3.5	0.96-7.1	47	2.4	0.74-5.3
U-Ca (mmol/h)	86	0.21	0.05-0.41	39	0.25	0.08-0.41	47	0.19	0.05-0.34
U-Ca (mmol/24h)	86	5.1	1.3-9.8	39	5.9	1.8-9.8	47	4.5	1.3-8.1
U-Ca (mmol/mmol creatinine)	86	0.43	0.14-1.4	39	0.42	0.14-0.79	47	0.50	0.17-1.4
**Overnight sample**									
U-Ca (mmol/l)	105	3.4	0.64-15	47	4.2	0.67-11	58	3.1	0.64-15
U-Ca (mmol/h)	105	0.15	0.03-0.4	47	0.18	0.04-0.41	58	0.13	0.03-0.30
U-Ca (mmol/mmol creatinine)	105	0.31	0.06-1.1	47	0.31	0.07-0.67	58	0.32	0.06-1.06
**Serum**									
S-Ca, ionized (mmol/l)	107	1.2	1.0-1.4	48	1.2	1.2-1.3	59	1.2	1.0-1.4
S-25(OH)D3 (ng/ml)	98	25.5	8.1-48.9	45	24.1	11.5-48.9	53	26.3	8.1-46.1
S-1.25(OH)_2_D3 (pg/ml)	98	33.8	13.7-97.1	45	33.0	18.6-97.1	53	34.9	13.7-70.8
S-PTH (ng/l)	106	38.4	14.8-398	48	43.6	20.6-398	58	36.7	14.8-91.6

Table 1 also shows serum levels of ionized Ca, parathyroid hormone, 25(OH)D3, and 1,25(OH)_2_D3, together with the urinary calcium excretion in 24-hour samples and timed overnight samples. The excretion of U-Ca per hour was significantly higher in the 24 hour-samples than in the timed morning sample (p < 0.001). This difference was found also after normalizing for creatinine (p < 0.001). There was a positive correlation between the excretion of U-Ca (mmol/h) and urinary creatinine (mmol/h) (r_p_ = 0.45, p < 0.001 for the 24-hour sample and r_p_ = 0.35, p < 0.001 for the timed overnight sample). In overnight urine, there was also a significant positive correlation between diuresis (urinary flow rate mL/h) and U-Ca (r_p_ = 0.30, p = 0.002) and creatinine (r_p_ = 0.28, p = 0.004) excretion rates. Men excreted significantly more U-Ca per hour than women, both in the 24-hour sample (p < 0.001) and in the timed overnight urine sample (p = 0.005). After normalizing for creatinine, there was instead a trend for women to excrete more U-Ca than men (Table 1).

U-Ca excretion (excretion rates per hour and levels normalized for creatinine) was positively correlated with ionized S-Ca (Table 2). U-Ca normalized for creatinine was positively correlated with age and menopause (defined as age >51 years in women). Weight was positively associated with U-Ca excretion per hour, but negatively correlated with U-Ca normalized for creatinine (Table 2).

**Table 2:** Correlations between parameters (Pearson’s correlation coefficients)

	**K-Cd (μg/g ww)**	**Age**	**Sex**	**Weight**	**Menopause**	**S-Ca, ionized (mmol/l)**	**24-h urinary flow rate (ml/h)**	**Overnight urinary flow rate (ml/h)**	**S-25(OH)D3 (ng/ml)**	**24-h U-Ca (mmol/h)**	**24-h U-Ca (mmol/mmol creatinine)**	**ON U-Ca (mmol/h)**	**ON U-Ca (mmol/mmol creatinine)**
K-Cd, μg/g ww		0.28**	0.24*	-0.27**	0.33**	**0.06**	**-0.09**	**0.09**	**0.14**	**-0.06**	0.27*	**0.05**	0.27**
Age	0.33**		**-0.10**	**-0.009**	0.44**	-0.22*	-0.29**	**0.08**	**0.13**	**0.006**	0.25*	**0.14**	0.23*
Sex				-0.57**	0.49**	**0.06**	**0.07**	**-0.12**	**0.09**	-0.41**	**0.16**	-0.27**	**0.15**
Weight	-0.10	0.04			-0.32**	**0.15**	**-0.09**	**0.06**	-0.22*	0.23*	-0.30**	**0.17**	-0.25*
Menopause	0.31*	0.77**		-0.08		**-0.13**	**-0.13**	**-0.03**	**0.08**	**-0.14**	0.29**	**0.05**	0.34**
S-Ca, ionized, mmol/l	0.17	-0.15		0.21	-0.22		**0.05**	**0.12**	**-0.06**	**0.21**	0.30**	0.22*	0.26**
24-h urinary flow rate, ml/h	-0.02	-0.25		-0.01	-0.23	0.06		0.43**	**0.13**	**-0.01**	**-0.06**	**-0.06**	**-0.07**
Overnight urinary flow rate, ml/h	0.25	-0.04		-0.11	0.05	0.15	0.52**		**0.02**	**0.005**	**0.06**	0.30**	**0.16**
S-25(OH)D3, ng/ml	0.07	0.05		-0.08	0.05	-0.003	0.19	0.08		**-0.22**	**-0.11**	**-0.01**	**0.08**
24-h U-Ca, mmol/h	0.29*	0.19		-0.10	0.17	0.24	0.11	0.08	-0.07		0.62**	0.63**	0.46**
24-h U-Ca, mmol/mmol creatinine	0.35*	0.36*		-0.26	0.28	0.31*	-0.03	0.14	-0.07	0.82**		0.47**	0.72**
ON U-Ca, mmol/h	0.37**	0.25		-0.19	0.33*	0.19	0.04	0.31*	0.25	0.56**	0.57**		0.77**
ON U-Ca, mmol/mmol creatinine	0.37**	0.29*		-0.27*	0.36**	0.25	-0.03	0.21	0.22	0.57**	0.70**	0.91**	

Upper right triangular part of the table: all (bold). Lower left: women.

ON = overnight sample. Menopause assumed for women aged >51 years. *p <0.05, **p <0.01.

### Association between kidney Cd and urinary Ca excretion

**Figure 1:**
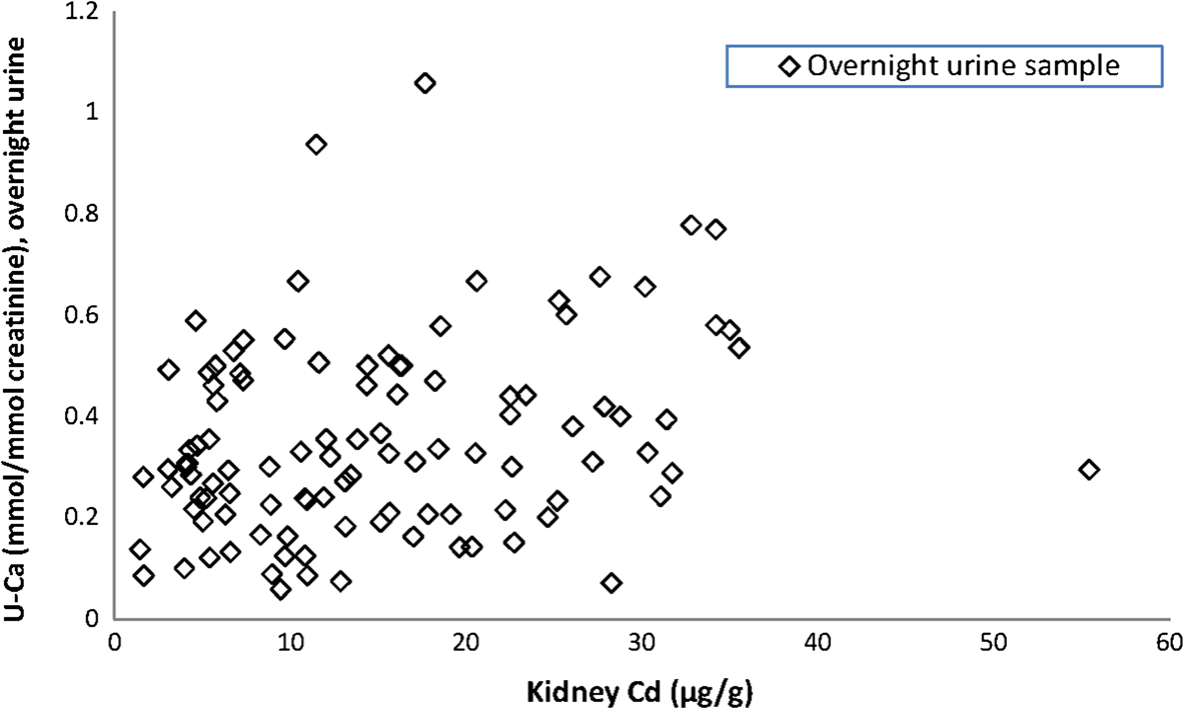
Excretion of urinary calcium (U-Ca) in overnight urine (mmol Ca/mmol creatinine) as a function of kidney cadmium (kidney Cd, μg/g wet weight) in living kidney donors (N = 105).

There was a significant positive correlation between continuous kidney Cd and urinary calcium normalized for creatinine (Figure 1), both in the 24-hour sample (r_s_ = 0.32, p = 0.003, r_p_ = 0.27, p = 0.01) and in the timed overnight sample (r_s_ = 0.27, p = 0.005, r_p_ = 0.27, p = 0.006), while there was no such association for Ca excretion per hour (Table 2). Likewise, in women kidney cadmium (as a continuous variable) correlated significantly to urinary calcium normalized for creatinine (24-hour sample p = 0.02; overnight sample p = 0.004) and urinary calcium excretion per hour (p = 0.049 and p = 0.004) (Table 2). In men, there were no such significant associations.

We also calculated associations between categorical K-Cd and U-Ca, dividing the donors in those above (“high K-Cd”) and equal to or below (“low K-Cd”) the median kidney Cd (12.9 μg/g) (mean high K-Cd 22.9, low K-Cd 7.3 μg/g). Donors with high kidney Cd had higher excretion of U-Ca normalized for creatinine than those with low kidney Cd (24 hour sample mean 0.53 vs. 0.39 mmol Ca/mmol creatinine, p < 0.001 (Additional file 1: Figure S2); overnight sample 0.40 vs. 0.31 mmol Ca/mmol creatinine, p = 0.02). This was mainly caused by a difference in women (p = 0.001 and p = 0.02), while there was no significant difference in men. In women, also the excretion of Ca per hour was higher in those with high kidney Cd (24 hour sample mean 0.21 vs. 0.15 mmol/h, p = 0.006; overnight sample 0.16 vs. 0.11 mmol/h, p = 0.01). When stratifying for smoking (ever/never-smokers), those with high kidney Cd had significantly higher 24-hour urinary calcium normalized for creatinine both in never-smokers (p = 0.009), and in ever-smokers (p = 0.03).

In multiple linear regression models, including categorical kidney Cd (high/low), age, sex, weight, menopause, ionized serum calcium, urinary flow rate, and vitamin D (25(OH)D3), urinary calcium excretion in the 24-hour sample was significantly associated with high kidney Cd (20% higher U-Ca mmol/h, p = 0.04, and 32% higher U-Ca normalized for creatinine, p = 0.002) (Table 3). There was, however, no such impact of kidney Cd in the overnight urine sample. The regression model was constructed after backward elimination and smoking, serum PTH and 1,25(OH)_2_D3 were excluded from the final model as these variables were not significantly associated with urinary calcium excretion. We repeated the multiple linear regression analysis for women and men separately. In women, again, high kidney Cd had a significant impact on U-Ca in the 24-hour urine sample (40% higher U-Ca mmol/h, p = 0.01, and 47% higher U-Ca normalized for creatinine, p = 0.003). In men, no effect of kidney Cd was found. When kidney Cd was treated as a continuous variable, the effect of kidney Cd was not statistically significant (p = 0.08 for U-Ca normalized for creatinine in 24-hour urine) (Additional file 2: Table S1 and S2).

**Table 3:** Multiple linear regression analysis with U-Ca as the dependent variable

	**24-h U-Ca (mmol/h)**	**24-h U-Ca (mmol/mmol creatinine)**	**ON U-Ca (mmol/h)**	**ON U-Ca (mmol/mmol creatinine)**
R^2^	0.34	0.52	0.27	0.31
Intercept	-0.39	-2.1	-0.78	-2.1
*Regression coefficients*				
Categorical K-Cd (high/low)	0.04*	0.12**	-0.002	0.01
Age	-0.001	0.003	0.0004	0.003
Sex	-0.09**	-0.09	-0.05*	-0.04
Weight	-0.0004	-0.006**	0.0005	-0.004*
Menopause	0.04	0.09	0.04	0.13*
S-Ca, ionized, mmol/l	0.57*	2.4**	0.69**	2.05**
24-h urinary flow rate, ml/h	0.0007	0.001		
ON urinary flow rate, ml/h			0.0007*	0.0009
S-25(OH)D3, ng/ml	-0.003*	-0.006**	0.0002	0.0003

ON = overnight sample. Menopause assumed for women aged >51 years (coded 1 if present, otherwise 0). N = 76 observations used. *p < 0.05, **p < 0.01.

We also tested the impact of high kidney Cd on the odds of having high calcium excretion in the 24-hour sample, defined as the upper quartile of U-Ca (>0.56 mmol/mmol creatinine). In a stepwise (p = 0.1 for inclusion in the model) logistic regression model high kidney Cd increased the odds of having high U-Ca normalized for creatinine (OR 5.5, 95% CI 1.6-19). The only other variable included in the final model was body weight (Additional file 2: Table S3). When stratifying for sex, the risk estimate was higher for women; OR 8.9 (95% CI 1.6-49) for having high U-Ca normalized for creatinine. For men, the OR was 2.4 (95% CI 0.4-16) but not statistically significant. There was no significant effect of high kidney cadmium on the odds of having high calcium excretion per hour (Additional file 2: Table S3).

In multivariate analyses with either serum ionized Ca, parathyroid hormone, or inactive or active vitamin D3 as the dependent variable, we found no significant associations with kidney Cd (dichotomized or continuous). In women, there was however a tendency towards an association between dichotomized kidney Cd and ionized S-Ca (p = 0.053).

### Association between kidney Cd and bone mineral density

High kidney Cd (above median) was negatively associated with BMD for total body, spine, femur and forearm. However, in multiple linear regression analyses, including possible confounders and effect modifiers in the model (age, sex, body weight, menopause, smoking and vitamin D), we found no significant associations between kidney Cd and BMD for any location (Additional file 2: Table S4). For total body and femur, weight was the only factor significantly associated to BMD in the model. Forearm BMD was associated with sex as well (lower in women). Smoking was the only factor in the model significantly associated with BMD of the spine.

In group comparison, smokers with high kidney Cd had lower BMD for total body, spine, femur and forearm than those with low kidney Cd, but the differences were not statistically significant (p = 0.06 for total body, Additional file 3: Figure S3), except for BMD of the femoral trochanter (p = 0.049). In those aged > 51 years, BMD for total body, the femoral trochanter and part of the forearm was significantly lower in the group with high kidney Cd (p < 0.05, Additional file 3: Figure S3). The difference was not significant for the younger group.

When the multivariate analyses were repeated and stratified for sex or age, the only statistically significant association between kidney Cd (high/low) and BMD was in the forearm in those aged ≤ 51 years (p = 0.03). An analysis stratified for smoking showed no significant associations between BMD and kidney Cd.

There were significant negative correlations between BMD and urinary calcium normalized for creatinine for most sites. However, in a multiple regression model including age, sex, body weight, menopause, smoking and vitamin D, these associations were no longer significant.

## Discussion

This study is the first to report significant positive associations between kidney Cd levels in biopsies from living donors and urinary calcium excretion. We observed that donors with high kidney Cd (above the median) had significantly higher U-Ca excretion normalized for creatinine than those with low kidney Cd, mainly due to a significant difference in women but not men. We also found a significant positive association between U-Ca excretion in the 24-hour sample and kidney Cd in a multiple linear regression model, including age, sex, weight, menopause, ionized serum calcium, urinary flow rate and vitamin D (25(OH)D3). We believe that the association was stronger in 24-hour urine because that sample was collected during a longer time than the overnight sample, and should therefore give a more reliable measure of the mean urinary calcium concentration.

Men excreted significantly more U-Ca per hour than women, in accordance with previous studies [25]. However, after normalizing for creatinine (Ca/creatinine ratio), there was a trend for women to excrete more calcium than men. The tendency towards stronger associations between kidney Cd and U-Ca when using dichotomized instead of continuous kidney Cd could indicate a threshold effect, very low kidney cadmium levels not affecting U-Ca.

Cadmium is known to affect both kidney function and the skeleton, as was first described as the Itai-Itai disease in Japan [2]. Later studies have confirmed an increased risk for osteoporosis and/or fractures after Cd exposure [8-13]. We focused on the relationship between kidney Cd and urinary calcium excretion, but we also measured BMD in part of the study population. The effect of Cd on calcium metabolism is interesting as urinary calcium is a marker of tubular function, but also because calcium is the most important mineral in the bone tissue. Some previous studies have shown a positive association between urinary Cd, as a marker of Cd body burden, and calcium excretion [17,19]. However, urinary Cd is not an ideal indicator of the body burden because Cd excretion may increase when kidney function is impaired, and Cd may be co-excreted with urinary proteins [21,26,27]. One of the strengths with this study is the use of kidney Cd, which eliminates the risk of a non-causal association due to diuresis or co-excretion of Cd and protein.

The effect of kidney Cd on urinary calcium excretion was mainly seen in women. This is supported by a study of residents in Thailand with elevated urinary Cd, where the fractional excretion of calcium increased with rising cadmium exposure (urinary Cd) especially in women [28]. In that study, there was also a positive correlation between fractional calcium excretion and the excretion of bone resorption markers, and the levels of those markers were significantly higher in women. This could indicate that women are at higher risk for bone effects resulting from cadmium exposure than men.

The mechanism behind the increased urinary calcium excretion in donors with high kidney Cd in this study is not clear, but it could be an effect of early renal tubular dysfunction [17] or a direct effect on bone, possibly by increased bone resorption [2,5,12,14,15,20]. A third possibility is that calcium deficiency, following calcium loss in the urine, has led to an increased expression of essential metal transporters in the intestine, thereby also increasing the absorption of cadmium [6,7]. An increased calcium excretion can be both the cause of, and an effect of, increased bone resorption, which can result in low BMD (osteopenia or osteoporosis) and increased risk of fractures [10,11,13].

In the present study, we found significant negative correlations between kidney Cd and BMD in the univariate analyses, but no significant associations in the multiple regression analyses, where body weight was the predominant factor and positively associated to BMD. Other possible confounders or effect modifiers included in the model were age, sex, menopause, smoking and vitamin D. The absence of significant associations could indicate that the increased Ca excretion is not associated with low BMD at these low Cd levels. However, with BMD measured in only 67 of the 109 donors with data on kidney Cd levels, our small group size provides limited power to find an association between kidney Cd and BMD. In addition, most of our subjects may have been too young to have an effect of Cd on BMD (median age 51 years).

Increasing age, female gender, estrogen deficiency, e.g. at menopause, low BMI and weight, osteoporosis in the family history, smoking and previous fractures are known risk factors for low BMD [29]. There is also a positive association between physical activity and bone mass. We had no information about menopausal age, osteoporosis in the family or level of physical activity, which is a limitation of the study. However, menopause was assumed for women aged >51 years, which is the median age for menopause [24]. We also had no information about dietary calcium intake, which may contribute to variability in calcium levels. Vitamin D is usually affected by season, which was confirmed in this study (data not shown). These sources of variability in covariates could lead to some misclassification, but such misclassification should be non-differential.

The biopsies in the study were taken from living kidney donors. In other circumstances it would not be ethical to take kidney biopsies from healthy people, but at this transplantation center a “baseline” biopsy is normally taken from the donated kidney, as part of the routine at transplantation. Thus the study group does not include subjects with diabetes or other serious systemic diseases. However, we consider them representative for the healthy part of the general population.

## Conclusions

In the present study, we found significant positive associations between urinary calcium excretion and kidney cortex Cd in healthy subjects, mainly women, in spite of rather low kidney Cd (median 12.9 μg/g). This relation between kidney Cd and urinary calcium provides some support for the results from other studies, which have shown an effect on calcium excretion and kidney function at low levels of urinary Cd [2,19], especially a study from Thailand showing increased fractional excretion of calcium with rising cadmium exposure particularly in women [28]. The results from our study somewhat strengthen the case for preventive measures against Cd pollution, although more research is needed to explain the mechanisms of the effects of Cd on bone and calcium metabolism.

## Abbreviations

25(OH)D3: 25-hydroxyvitamin D or calcidiol;1,25(OH)2D3: 1,25-dihydroxyvitamin D or calcitriol;BMD: Bone mineral density;Ca: Calcium;Cd: Cadmium;EFSA: European Food Safety Authority;K-Cd: Kidney cadmium;MT: Metallothionein;PTH: Parathyroid hormone;U-Ca: Urinary calcium;ww: Wet weight

## Competing interests

All authors state that they have no conflicts of interest.

## Authors’ contributions

Study design: GS, EFL, LB. Study conduct: GS, EFL, TL, LB. Data collection: MW, GS, EFL, TL, LB. Data analysis: MW, GS, CÖ, LB. Data interpretation: MW, GS, CÖ, LB. Drafting manuscript: MW, GS, LB. Revising manuscript content: MW, GS, LB. Approving final version of manuscript: MW, GS, EFL, CÖ, TL, LB. MW, GS and LB take the responsibility for the integrity of the data analysis. All authors read and approved the final manuscript.

## Supplementary Material

Additional file 1: Figure S2 Mean excretion of urinary calcium (U-Ca) in 24-hour urine (mmol Ca/mmol creatinine) at kidney cadmium below or above the median (low/high kidney Cd) in living kidney donors (N = 86). Click here for file

Additional file 2: Table S1 Multiple linear regression analysis with U-Ca as the dependent variable. **Table S2.** Multiple linear regression analysis with U-Ca as the dependent variable (women). **Table S3.** Multiple logistic regression analysis with high U-Ca (upper quartile) as the dependent variable. **Table S4.** Multiple linear regression analysis with BMD as the dependent variable. Click here for file

Additional file 3: Figure S3 Mean bone mineral density (BMD), total body, at kidney cadmium below or above the median (low/high kidney Cd) in living kidney donors stratified for smoking (ever/never) and age (≤51 or >51 years). Click here for file
