# Supplementary figures and images for: Kidney cadmium levels and associations with urinary calcium and bone mineral density: a cross-sectional study in Sweden
Source: Environ Health. 2013 Mar 7;12:22. doi: 10.1186/1476-069X-12-22 (PMC3627629; doi:10.1186/1476-069X-12-22)

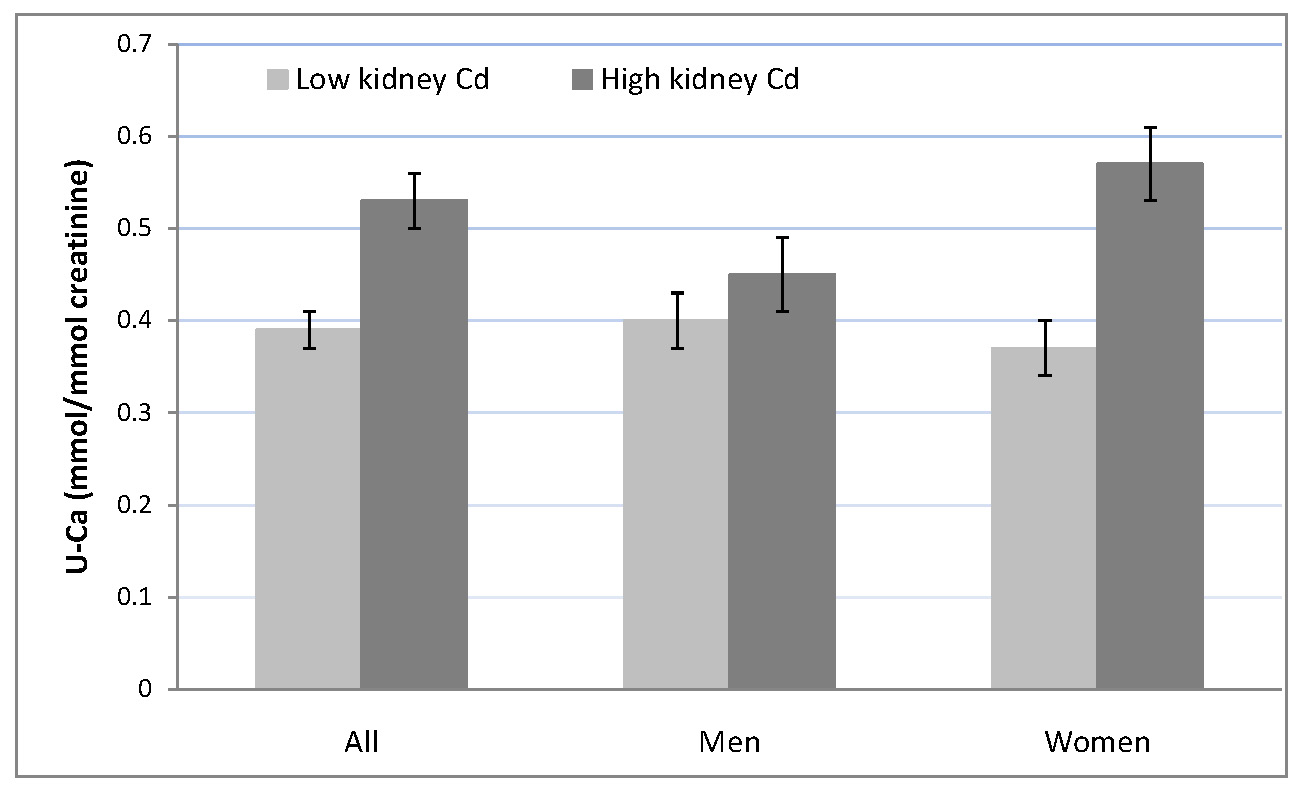

Supplement: Additional file 1: Figure S2 — Mean excretion of urinary calcium (U-Ca) in 24-hour urine (mmol Ca/mmol creatinine) at kidney cadmium below or above the median (low/high kidney Cd) in living kidney donors (N = 86). [file 1476-069X-12-22-S1.jpeg]

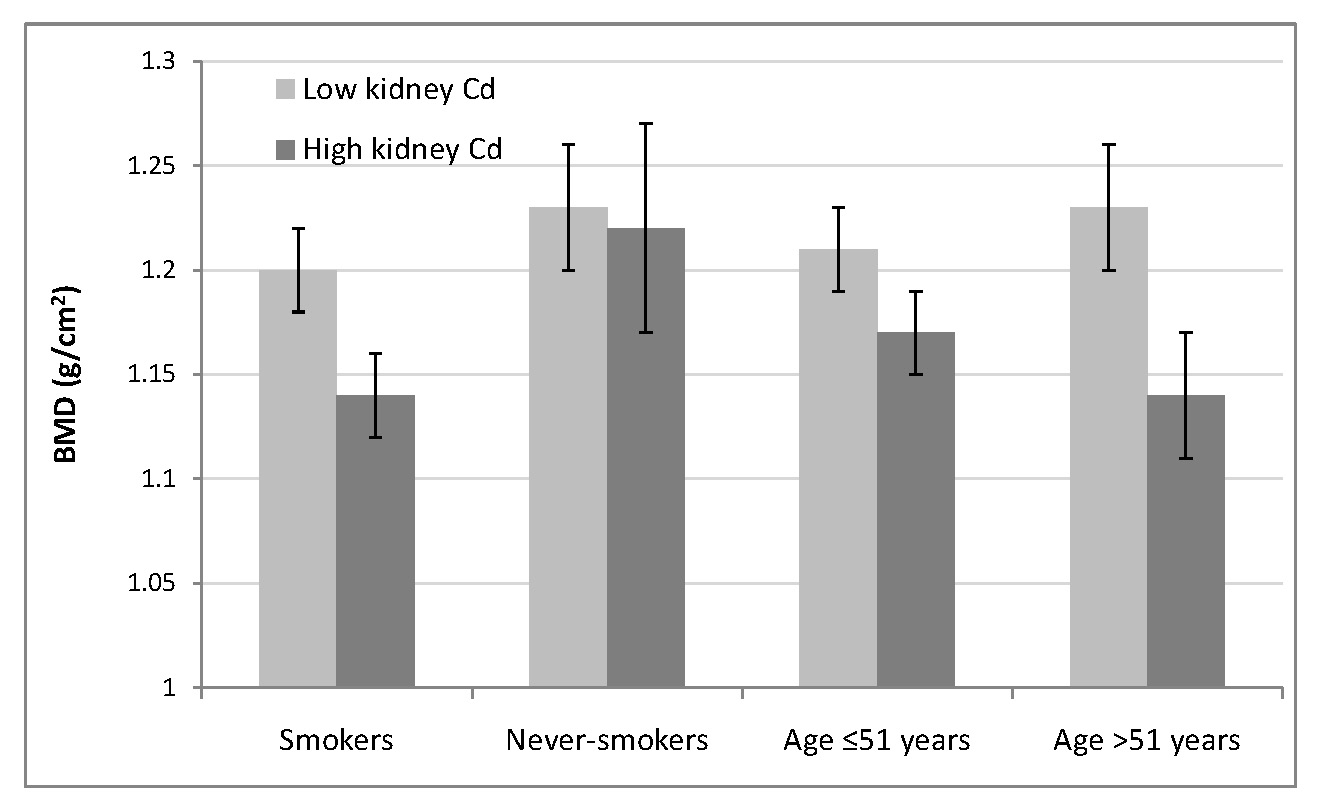

Supplement: Additional file 3: Figure S3 — Mean bone mineral density (BMD), total body, at kidney cadmium below or above the median (low/high kidney Cd) in living kidney donors stratified for smoking (ever/never) and age (≤51 or >51 years). [file 1476-069X-12-22-S3.jpeg]
