# Supplementary material for: Kidney cadmium levels and associations with urinary calcium and bone mineral density: a cross-sectional study in Sweden
Source: Environ Health. 2013 Mar 7;12:22. doi: 10.1186/1476-069X-12-22 (PMC3627629; doi:10.1186/1476-069X-12-22)
Supplement: Additional file 2: Table S1 — Multiple linear regression analysis with U-Ca as the dependent variable. Table S2. Multiple linear regression analysis with U-Ca as the dependent variable (women). Table S3. Multiple logistic regression analysis with high U-Ca (upper quartile) as the dependent variable. Table S4. Multiple linear regression analysis with BMD as the dependent variable. [file 1476-069X-12-22-S2.docx]

**Additional file 2:**

**Table S1. Multiple linear regression analysis with U-Ca as the dependent variable**

|  | 24-h U-Ca  (mmol/h) | 24-h U-Ca  (mmol/mmol creatinine) | ON U-Ca  (mmol/h) | ON U-Ca  (mmol/mmol creatinine) |
| --- | --- | --- | --- | --- |
| R² | 0.32 | 0.47 | 0.27 | 0.31 |
| Intercept | -0.49 | -2.41 | -0.77 | -2.1 |
|  |  |  |  |  |
| *Regression coefficients* |  |  |  |  |
| Continuous K-Cd (µg/g ww) | 0.001 | 0.004 | 0.00009 | 0.002 |
| Age | -0.0008 | 0.004 | 0.0003 | 0.002 |
| Sex | -0.09** | -0.10 | -0.05* | -0.04 |
| Weight | -0.0005 | -0.007** | 0.0006 | -0.003* |
| Menopause | 0.04 | 0.08 | 0.04 | 0.13* |
| S-Ca, ionized, mmol/l | 0.66** | 2.6** | 0.68** | 2.04** |
| 24-h urinary flow rate, ml/h | 0.0006 | 0.0008 |  |  |
| ON urinary flow rate, ml/h |  |  | 0.0007* | 0.0009 |
| S-25(OH)D3, ng/ml | -0.002* | -0.005* | 0.0002 | 0.0003 |

ON=overnight sample. Menopause assumed for women aged >51 years (coded 1 if present, otherwise 0). N=76 observations used. *p<0.05, **p<0.01.

**Table S2. Multiple linear regression analysis with U-Ca as the dependent variable (women)**

|  | 24-h U-Ca  (mmol/h) | 24-h U-Ca  (mmol/mmol creatinine) | ON U-Ca  (mmol/h) | ON U-Ca  (mmol/mmol creatinine) |
| --- | --- | --- | --- | --- |
| R² | 0.28 | 0.54 | 0.31 | 0.41 |
| Intercept | -0.53 | -2.87 | -0.63 | -2.3 |
|  |  |  |  |  |
| *Regression coefficients* |  |  |  |  |
| Continuous K-Cd (µg/g ww) | 0.002 | 0.005 | 0.0007 | 0.003 |
| Age | 0.0005 | 0.006 | -0.0002 | 0.001 |
| Weight | -0.001 | -0.01** | -0.0006 | -0.006* |
| Menopause | 0.02 | 0.04 | 0.04 | 0.14 |
| S-Ca, ionized, mmol/l | 0.57 | 3.00** | 0.57* | 2.27** |
| 24-h urinary flow rate, ml/h | 0.0009 | 0.001 |  |  |
| ON urinary flow rate, ml/h |  |  | 0.0007 | 0.001 |
| S-25(OH)D3, ng/ml | -0.001 | -0.003 | 0.002 | 0.004 |

ON=overnight sample. Menopause assumed for women aged >51 years (coded 1 if present, otherwise 0). N=49 observations used. *p<0.05, **p<0.01

**Table S3. Multiple logistic regression analysis with high U-Ca (upper quartile) as the dependent variable**

|  | 24-h U-Ca, upper quartile  (mmol/h) | 24-h U-Ca, upper quartile  (mmol/mmol creatinine) |
| --- | --- | --- |
| Intercept | -4.07 | 2.06 |
| *Regression coefficients* |  |  |
| Categorical K-Cd (high/low) | 0.24 | 1.71* |
| Body weight | 0.03 | -0.06 |

*OR=5.5 (1.6-18.6)

**Table S4. Multiple linear regression analysis with BMD as the dependent variable**

|  | BMD total body | BMD spine | BMD femur (neck) | BMD forearm |
| --- | --- | --- | --- | --- |
| R² | 0.53 | 0.29 | 0.31 | 0.60 |
| Intercept | 0.99 | 1.17 | 0.77 | 0.26 |
|  |  |  |  |  |
| *Regression coefficients* |  |  |  |  |
| Categorical K-Cd (high/low) | -0.02 | -0.01 | 0.01 | -0.02 |
| Age | 0.002 | 0.001 | -0.0002 | 0.001 |
| Sex | -0.04 | -0.02 | -0.02 | -0.05* |
| Weight | 0.003** | 0.002 | 0.003* | 0.002* |
| Menopause | -0.05 | -0.11 | -0.02 | -0.04 |
| Smoking | -0.04 | -0.15* | -0.02 | -0.001 |
| S-25(OH)D3, ng/mL | -0.001 | -0.003 | -0.002 | -0.00002 |

Menopause assumed for women aged >51 years (coded 1 if present, otherwise 0). *p<0.05, **p<0.01
